# The Effect of the Gutta Condensor on Powder-Liquid and Ready-to-Use Hydraulic Sealers

**DOI:** 10.4317/jced.63891

**Published:** 2026-06-29

**Authors:** Gabriel da Siqueira Felske, Gabriel Barcelos Só, Carolina Horn Troian Michel, Ricardo Abreu da Rosa, Marco Antônio Húngaro Duarte, Jefferson Ricardo Pereira, Marcus Vinicius Reis Só

**Affiliations:** 1 Department of Conservative Dentistry, School of Dentistry, Rio Grande do Sul Federal University (UFRGS), Porto Alegre, RS, Brazil; 2 Department of Stomatology, Faculty of Dentistry, Federal University of Santa Maria (UFSM), Santa Maria, RS, Brazil; 3 Department of Operative Dentistry, Endodontics and Dental Materials, Bauru School of Dentistry, University of São Paulo, São Paulo, Brazil; 4 Department of Prosthodontics, Unisul - Universidade do Sul de Santa Catarina, Tubarão, SC, Brazil

## Abstract

**Background:**

The purpose of this study was to evaluate the increase in internal and external temperature of bovine tooth roots by driving the Gutta Condensor at two different speeds, as well as whether the sealers activation can cause alterations in the chemical structure of Bio-C Sealer and Cimmo HP, through Raman spectroscopy.

**Materials and Methods:**

To assess the variation in root temperature, 60 bovine teeth were used. They were sectioned to standardize the samples to a length of 12mm, and their canals were prepared using VDW R50. After preparation, the roots were distributed into 12 groups, based on sealer type, use or non-use of gutta-percha, and Gutta Condensor speed. During root canal filling, internal and external temperatures of the roots were measured in all groups. To access the chemical structure, Raman spectroscopy was performed on both thermally activated and non-activated sealer samples (n=2).

**Results:**

It was demonstrated that both the sealer and the gutta-percha influenced the increase in internal and external root temperature. In intragroup comparisons, differences were observed between the speed of the Condensor and the control samples (P &lt; 0.05). Raman spectroscopy showed that both control and activated samples have the same chemical structure.

**Conclusions:**

This study concluded that the Gutta Condensor increases both internal and external temperatures to a non-critical point but does not affect the chemical structure of the sealer.

## Introduction

The success of endodontic treatment is linked to effective cleaning of the root canal system and to a hermetic, three-dimensional filling of the root canals, as this sealing prevents bacterial and oral fluid infiltration, thereby ensuring periapical health (1). Root canal filling is commonly performed using a combination of endodontic sealers and gutta-percha. Gutta-percha alone does not adhere to the dentinal walls (2); thus, endodontic sealers are employed to fill the voids between gutta-percha and the canal walls (3). There are several techniques for filling root canals, such as lateral condensation, which is widely used (4). Later, thermoplasticization techniques for gutta-percha were introduced to better fill difficult-to-access areas with gutta-percha cones and sealers (5). In the thermomechanical compaction technique, the compactor is activated, generating heat through friction and softening gutta-percha, allowing a three-dimensional filling of the root canal (6). Hydraulic sealers, commonly known as bioceramic sealers, are composed of inorganic oxide and silicate particles, such as calcium silicate, aluminum silicate, and silicon phosphate, combined with an activating liquid (di- and tricalcium silicate, bismuth oxide, zirconium oxide) (7). These sealers set through a hydration reaction; when in contact with moisture, the sealer dissociates into calcium hydroxide, which induces the formation of hydroxyapatite on its surface (7). For this reason, they are considered bioactive materials, exhibiting antimicrobial properties, the ability to induce tissue regeneration, and high biocompatibility (8). Earlier studies evaluated heat transfer during thermoplastic filling to the external root surface or simulated periodontal ligament using non-bioceramic sealers. Following the standard established by (9), a temperature rise of 10°C above body temperature (37°C) was observed to cause injury to the periodontal ligament. A critical factor when selecting a sealer is therefore the amount of heat it will be exposed to during obturation (10). The use of heat during root canal filling makes it easier to fill hard-to-reach areas. To date, no study has evaluated the effect of the condensor on root temperature when using bioceramic sealers. This study aimed to evaluate the increase in internal and external root temperature of bovine teeth when using the Gutta Condensor at two speeds and two different bioceramic sealers, one ready-for-use (Bio-C Sealer - Angelus, Londrina PR, Brazil) and one powder-liquid (Cimmo HP - Cimmo, Pouso Alegre, MG, Brazil), alone or in combination with gutta-percha, and the effect of their activation on the chemical structure through Raman spectroscopy. The null hypotheses are that there is no change in internal and external root temperature, regardless of the speed of the Gutta Condensor, the sealer, or the use of gutta-percha, and that there is no change in the chemical structure of the tested sealers, regardless of rotation speed.

## Materials and Methods

- Study Design This is a controlled in vitro study. The factors under investigation are possible temperature changes at the root surface (Part 1) and the chemical structures of two bioceramic endodontic sealers (Part 2) when a Gutta Condensor is used. PART 1. Root temperature. - Ethical Considerations This study was submitted to the Research Committee of the Faculty of Dentistry at UFRGS (COMPESQ). It was conducted in accordance with the Law on Procedures for the Scientific Use of Animals - Law No. 11,794 (10/08/2008) and the guidelines of the Ethics Committee on the Use of Animals (CEUA) of the Federal University of Rio Grande do Sul. - Sample Collection and Preparation For the sample size calculation regarding the evaluation of external root temperature, the following parameters were considered (11): Statistical test: ANOVA and Tukey test; Minimum difference between treatment means = 0.84; Standard deviation of error = 0.295; Number of treatments = 12; Power of the test = 0.8; Significance level = 0.05; Number of repetitions per treatment = 5. The sample consisted of 60 bovine teeth. Teeth were excluded if they presented: a) length less than 12 mm; b) root fractures or cracks detected by visual examination and with the aid of a 10x magnifying lens; c) incomplete apex formation; d) internal or external resorptions; e) foraminal opening larger than #45; f) foraminal diameter smaller than #25. Initially, the selected teeth were sterilized in an autoclave at 121°C for 15 minutes. Then, they were fixed in an acrylic plate with Type I Godiva (Kerr Co., Romulus, MI, USA) and were sectioned under irrigation with distilled water using an ISOMET Low-Speed Saw metallographic machine (Buehler Ltd., Lake Bluff, IL, USA), ensuring the sample length was standardized to 12 mm. Before chemical-mechanical preparation, each root canal was explored with hand K-type endodontic files (Dentsply-Maillefer, Ballaigues, Switzerland) ranging from #25 to #45 to determine the foraminal diameter. The teeth with foraminal diameters smaller than #25 and larger than #45 were excluded from the sample. The working length was visually set 1 mm short of the apical foramen, resulting in a length of 11 mm. Roots were distributed into groups following a stratified randomization. Stratification was based on canal diameter before preparation to avoid significant differences in canal volume between groups. After group allocation, chemical-mechanical preparation was performed using a reciprocating instrument R50 (VDW) powered by an electric motor (Reciproc Gold, VDW) under reciprocating motion. During each withdrawal of the instrument from the canal, irrigation with 5 ml of 2.5% sodium hypochlorite solution (Marcela, Porto Alegre, Brazil) was performed. At the end of preparation, the final irrigation protocol consisted of 3 ml of 17% EDTA (Marcela, Porto Alegre, Brazil) for 3 minutes, followed by irrigation with 5 ml of saline solution. Then, the canals were dried with two #45 absorbent paper points. - Experimental Groups Eight experimental groups were formed based on the bioceramic sealer, the use of a gutta-percha cone, and according to the speed of the Gutta Condensor. In addition, four control groups were established, bringing the total to 12. The root canals of four groups were filled with gutta-percha cones and Bio-C Sealer or Cimmo HP Sealer, using the Gutta Condensor at 6,000 RPM or 10,000 RPM (Endo Pro Torque, er, Carapicuíba, SP) for 10 seconds. In four other groups, root canals were filled without a gutta-percha cone, using only Bio-C Sealer or Cimmo HP Sealer and the Gutta Condensor at 6,000 RPM or 10,000 RPM for 10 seconds. In two groups, root canals were filled with sealer and a gutta-percha cone without thermocompaction; in two other groups, only with sealer, without thermocompaction. Each group (n=5) was named as follows: Experimental Groups: BC6CG: Bio-C Sealer + R50 Cone + Gutta Condensor 6,000 RPM BC10CG: Bio-C Sealer + R50 Cone + Gutta Condensor 10,000 RPM BC6SG: Bio-C Sealer + Gutta Condensor 6,000 RPM BC10SG: Bio-C Sealer + Gutta Condensor 10,000 RPM CM6CG: Cimmo HP + R50 Cone + Gutta Condensor 6,000 RPM CM10CG: Cimmo HP + R50 Cone + Gutta Condensor 10,000 RPM CM6SG: Cimmo HP + Gutta Condensor 6,000 RPM CM10SG: Cimmo HP + Gutta Condensor 10,000 RPM Control Groups: BCCNCG: Bio-C Sealer + R50 Cone BCCNSG: Bio-C Sealer CMCNCG: Cimmo HP + R50 Cone CMCNSG: Cimmo HP For root canal filling, all teeth that received gutta-percha cones were filled using the Hybrid Tagger technique. Initially, the master cone (R50, VDW) was tested for adaptation along the entire canal length. Then, endodontic sealers were handled according to the manufacturers' recommendations. The sealer was introduced into the root canal using a #1 Lentulo spiral placed at the canal orifice and activated at low speed. The procedure was repeated until complete canal filling was achieved for all groups. In groups using gutta-percha cones, the cone was inserted and advanced to the full canal length, followed by two accessory B8 gutta-percha cones using a two-digit spreader. In the experimental groups that received gutta-percha cones, a size #60 compactor was used to thermocompact the gutta-percha and sealer. In the experimental groups in which the root canals were filled with sealer only, the sealer was thermally activated with the same size #60 compactor. It was used with penetration and withdrawal movements along the material and canal walls, up to 8 mm in length, to plasticize the gutta-percha and activate the sealer. During and after use of the Gutta Condensor, the following tests were performed on the experimental and control groups. - Temperature Control To measure external and internal root temperature during Gutta Condensor use, a thermometer device (Minipar, APPAMT-520) was used. Temperature measurements were taken in three regions: the external root region of the cervical and middle third, and the internal region after using the thermocompactor. For external measurements, two cavities were drilled in each root-one in the cervical third and the other in the middle third-using a 1016 diamond bur to a depth of half the active tip. The thermometer was fixed in the cavities with fixing adhesive SuperBonder® (Loctite, Henkel Ltd., São Paulo). External temperature measurements were taken from the moment the compactor was activated until it was removed from the root canal. Internal temperature measurements were taken immediately after the compactor was removed. For all the procedures, teeth were fixed in a metal clamp with utility wax, and the ambient temperature was standardized at 17°C. Part 2. Sealers Chemical Structure. - Raman Spectroscopy Six small samples of each sealer were placed on a glass plate and handled according to the manufacturer's recommendations. Four of them were thermally activated by the Gutta Condensor for 10 seconds on the glass plate surface - two of them driven at 6,000 rpm, two at 10,000 rpm, and two remained unactivated (n=2). The chemical structures of the sealers at both compactor speeds were assessed using a Raman spectroscopy device (Senterra; Bruker Optics, Ettlingen, Germany). A 785 nm diode laser beam with 100 mW power, spectral resolution ~3.5 cm-1, and a fluorescence reduction filter was used, with the range set from 100 to 1400 cm-1. Twelve random points were read for 5 seconds each. The following bonds were observed: zirconium dioxide (ZrO2), dicalcium silicate (Ca2S), tricalcium silicate (Ca3S), and monoclinic zirconium dioxide (m-ZrO2). Spectra were analyzed using Opus 6.5 software (Bruker Optics, Germany). Spectral data (UA) were calculated and plotted along with other average spectra of the same sealer for comparison, performed by two examiners not involved in spectrum acquisition. - Statistical Analysis For root temperature, descriptive statistics, including mean and standard deviation, were calculated. The Shapiro-Wilk test was used to assess normal distribution. One-way ANOVA and Tukey tests were performed to analyze temperature control. For Raman data, a descriptive analysis of the observed phenomenon was conducted.

## Results

Regarding temperature variation, an intragroup comparison was initially performed based on the points at which temperature measurements were taken. The results are expressed in Table 1.

Table 1

For both Bio-C sealer and Cimmo HP, significantly higher temperatures were observed in all regions assessed when Condensor activation was performed at 10,000 rpm, with or without gutta-percha (P&lt;0.05). Activation with Condensor at 6,000 rpm had little impact on the increase in root temperatures, with significantly higher values only for the middle third with gutta-percha and cervical third without gutta-percha for the Bio-C sealer. For all other groups, there was no significant difference between the temperatures reached without activation and with activation at 6,000 rpm (P&gt;0.05). Table 2 presents the influence of gutta-percha on temperature rise across different measurement points.

Table 2

In the Bio-C Sealer groups, significantly higher temperatures were observed in the middle third and internal temperature for the group using gutta-percha and Gutta Condensor at 10,000 RPM (P &lt; 0.05). Meanwhile, in the Cimmo HP groups, significantly higher temperatures were observed across all analyzed regions when there was no gutta-percha and no Condensor activation (P &lt; 0.05). In Table 3, additional comparisons were conducted to determine whether sealer type influenced temperature rise under identical experimental conditions.

Table 3

**Figure 1:**
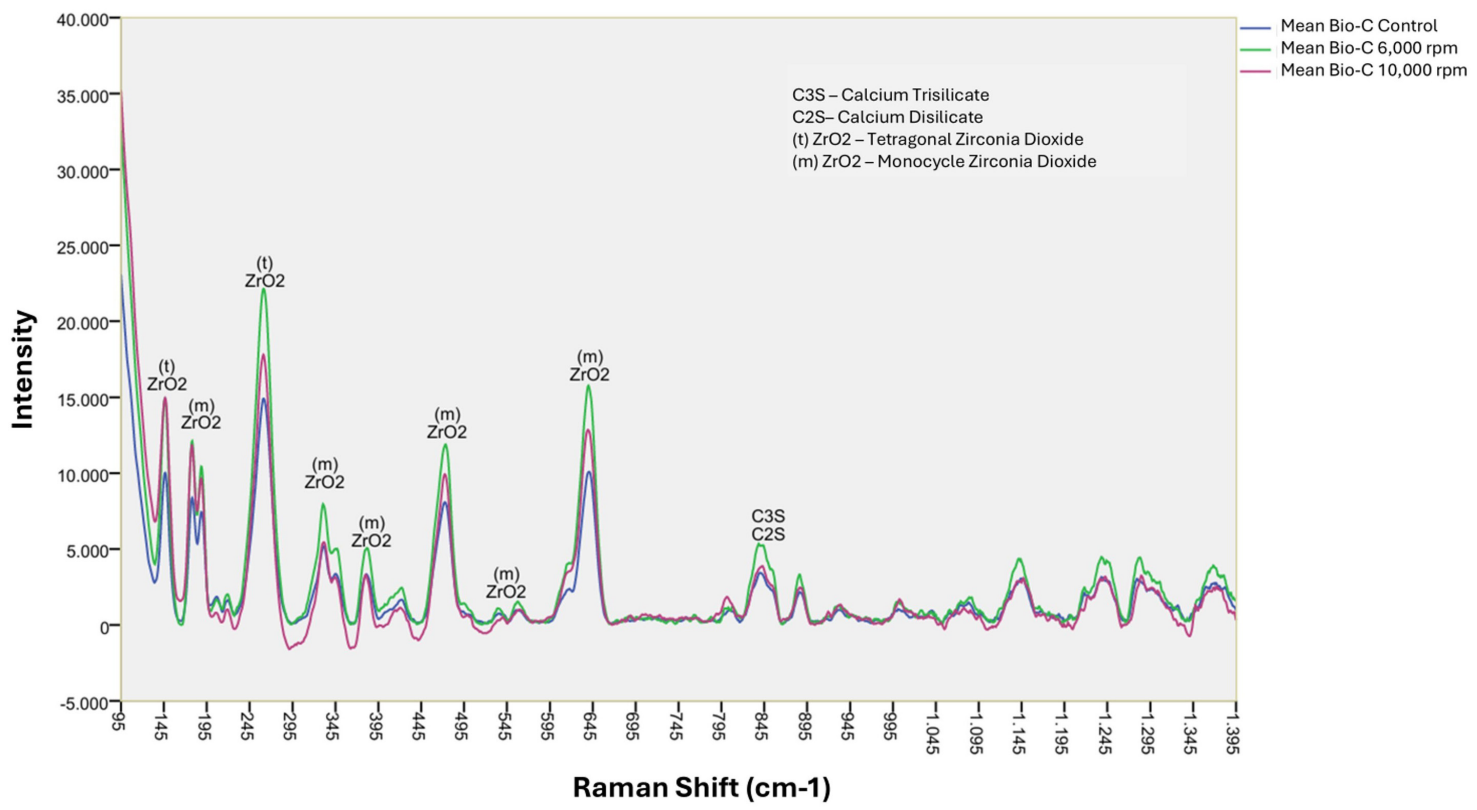
Raman spectroscopy of Bio C-Sealer with and without Condensor activation.

In the cervical and middle outer thirds, statistically significant differences were observed between control groups with and without gutta-percha, with higher temperatures for Cimmo HP. This difference was also detected in the internal root temperature without gutta-percha (P &lt; 0.005). When the fillings without guttapercha were activated at 6,000 rpm, there was a significant difference in the middle third and internally, with higher temperatures in Cimmo HP, while fillings with guttapercha activated at 10,000 rpm resulted in a significantly higher internal temperature with Bio-C Sealer (P &lt; 0.05). Regarding Raman spectroscopy, no alterations in chemical element peaks were observed in the generated spectra, regardless of the sealer form (ready-to-use or powder-liquid) or the compactor speed (6,000 or 10,000 RPM). The most representative bands observed for Bio-C sealer were calcium disilicate, calcium trisilicate, tetragonal zirconium dioxide, and monoclinic zirconium dioxide (Fig. 1).

1

**Figure 2:**
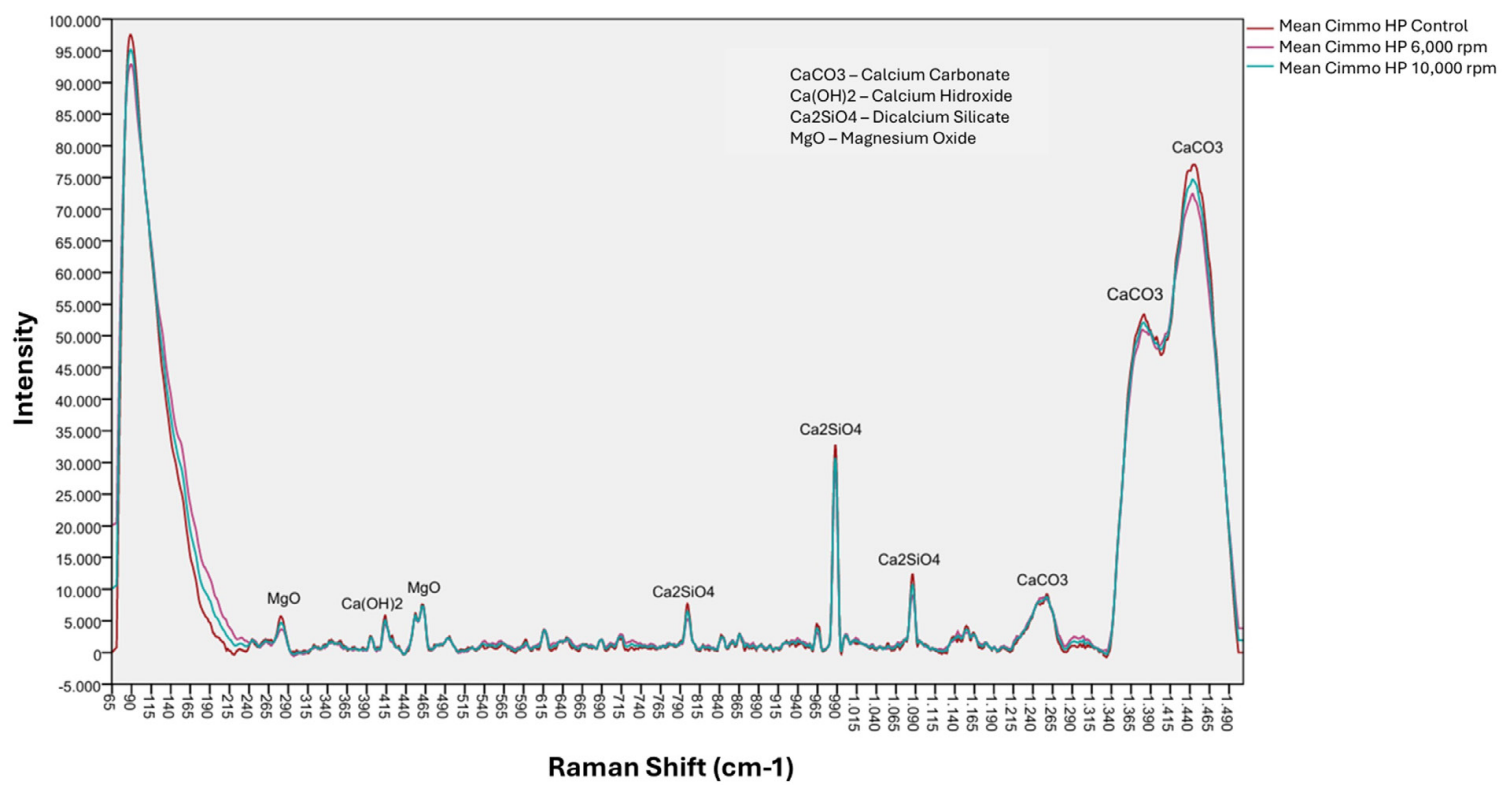
Raman spectroscopy of Cimmo HP Sealer with and without Condensor activation.

For Cimmo HP, the bands consisted of dicalcium silicate, calcium carbonate, calcium hydroxide, and magnesium oxide (Fig. 2).

2

## Discussion

This study was the first to investigate the impact of using the Gutta Condensor on root canal fillings with bioceramic sealers, both in relation to their chemical structure and the increase in root temperature. Currently, root canal filling techniques that thermoplastify gutta-percha demonstrate advantages in material adaptation to the root canal, providing improved three-dimensional sealing and mass homogeneity (5 , 6). One of the most widely used thermoplasticizing techniques is the Tagger hybrid. It provides a homogeneous filling, a good apical seal, and is easily accessible, as it only requires a low-speed contra-angle handpiece and a Gutta Condensor. However, the heat generated during thermoplasticization procedures has raised concerns (12). A systematic review investigated the effects of heating on bioceramic sealers and found that higher temperatures alter their physicochemical properties, including setting time, flow, and chemical composition; meanwhile, epoxy resin-based sealers remained stable under the same conditions. A critical factor when selecting a sealer is therefore the amount of heat it will be exposed to during obturation (10). In our study, the Gutta Condensor activation speed showed significantly higher average temperatures at 10,000 rpm across all evaluated sites, regardless of the sealer used. When activation was performed at 6,000 rpm, in general, there was no difference in temperature increase compared with the control groups. The presence of gutta-percha generally resulted in higher mean temperatures when associated with Condensor activation, likely due to friction with the compactor and subsequent heat generation. In the absence of activation, a different result was observed with the Cimmo HP sealer, showing significantly higher temperatures without gutta-percha. Likewise, when the two sealers were compared, Cimmo HP showed a tendency toward higher temperatures when gutta-percha was not used. This can be explained by differences in the presentation of the sealers: Cimmo HP is a powder-liquid, whereas Bio-C sealer is ready-to-use. The amount of sealer that fills the root canal in the absence of gutta-percha is greater. When a sealer with a powder-liquid presentation is used, its setting generates moderate heat due to a chemical hydration reaction. Measuring external root temperature in the cervical and middle thirds allows evaluation of heat transmission to the lateral periodontium. The increase in temperature on the external root surface can cause severe tissue injury. According to Ericksson et al., 1982 (9), a temperature 47°C is required to damage the periodontal ligament. In the present study, the maximum temperature recorded was 23.5°C. Previous studies using heated gutta-percha reported similar findings, with temperature increases below the critical threshold (13 - 15). Despite the increase in temperature observed when using thermoplasticization, no temperature reached using the Gutta Condensor would cause damage to the periodontal ligament, since the onset of tissue injury occurs at 47 degrees. Additionally, Raman spectroscopy confirmed that the tested bioceramic sealers maintained structural integrity under heat activation, in agreement with previous studies (16 - 18)-notably, long-term bioactivity results in the release of calcium ions and the formation of hydroxyapatite. This study has some limitations that should be considered. The first is that it was an in vitro study, meaning the results should not be directly extrapolated to a clinical scenario. The second is that it used bovine rather than human roots. Nevertheless, bovine roots allow for greater sample homogeneity in terms of dentin pattern, root size, and canal diameter (19). Finally, the third limitation is the use of a single thermoplasticization strategy for root canal filling. Additional studies testing the heating effects of other techniques, such as continuous-wave condensation, should be conducted to demonstrate their safety for clinical practice. The results of this study allow us to conclude that although the sealer influenced increases in root temperature, compactor speed, and the presence of gutta-percha, critical temperature values for periodontal tissue injury were not reached at any of the sites evaluated. Furthermore, the activation of the bioceramic sealers did not cause structural chemical changes.

## Conclusions

agdzdsfh

## Figures and Tables

**Table 1:** Table Means and standard deviation of the measured temperature of the samples, in °C. Intra-group analysis for each sealer (with and without gutta-percha and cervical, middle and internal).

		Cervical Outer third	Middle Outer third	Internal
With Gutta-Percha	BCCNCG	17.66 (0,1949) A	17,22 (0,1924) A	17,42 (0,1304) A
	BC6CG	18,34 (0,6107) A	18,6 (0,2915) B	18,28 (0,1789) A
	BC10CG	21,86 (0,6309) B	22,56 (0,7893) C	22,86 (1,09) B
WithoutGutta-Percha	BCCNSG	17,16 (0,1517) A	17,42 (0,5975) A	17,64 (0,4722) A
	BC6SG	18,96 (0,6504) B	18,54 (1,024) A	18,58 (0,3493) A
	BC10SG	20,82(1,062) C	21 (1,147) B	20,62 (1,011) B
With Gutta-Percha	CMCNCG	18,42 (0,4147) A	18,78 (0,497) A	17,54 (0,4722) A
	CM6CG	19,32 (0,7596) A	19,26 (0,6504) A	18,72 (0,719) A
	CM10CG	21,72 (0,563) B	21,5 (0,4505) B	21,1 (0,9925) B
WithoutGutta-Percha	CMCNSG	19,26 (0,5459) A	19,56 (0,2074) A	18,76 (0,3507) A
	CM6SG	19,6 (0,6285) A	20,12 (0,7463) A	19,52 (0,563) A
	CM10SG	20,62 (1,001) B	21,30 (0,9138) B	20,06 (0,6731) B

Equal capital letters in the column do not differ statistically by Tukey’s test (α = 5%).

**Table 2:** Table P values when evaluating the effect of gutta-percha on temperature increase.

		Cervical Outer third	Middle Outer third	Internal
BIO-C SEALER	CN	0,0856	0,4964	0,3447
	6000	0,1588	0,9028	0,1257
	10000	0,0964	0,0366*	0,0098*
CIMMO HP	CN	0,0255*	0,0119*	0,0017*
	6000	0,5431	0,088	0,0858
	10000	0,0646	0,6733	0,0884

*P values < 0.05 indicate statistically significant difference

**Table 3:** Table P values when evaluating the effect of the sealer on the temperature increase under the same experimental conditions.

	Cervical Outer third	Middle Outer third	Internal
BCCNCG vs. CMCNCG	0,0212*	0,0001*	0,9589
BCCNSG vs. CMCNSG	<0,0001*	<0,0001*	0,0014*
BC6CG vs. CM6CG	0,1324	0,4965	0,5173
BC6SG vs. CM6SG	0,4481	0,0160*	0,0389*
BC10CG vs. CM10CG	0,9934	0,2508	0,0452*
BC10SG vs. CM10SG	0,9814	0,9454	0,7909

*P values < 0.05 indicate statistically significant difference

## Data Availability

fhghgc
